# Erratum to: P89 Patient reported comorbidity is not a reliable data source: a systematic review of the literature

**DOI:** 10.1093/bjsopen/zrab071

**Published:** 2021-08-27

**Authors:** Chung Mun Alice Lin, Nathan Ng, Alexander Orman, Nicholas D Clement, David J Deehan

BJS Open, 5(Supplement_1), 2021, zrab032.088, https://doi.org/10.1093/bjsopen/zrab032.088

In the originally published version of this manuscript, there was an omission in the author list. The co-author “Nathan Ng” should have been included.

